# Application value of serum high mobility group protein B1 (HMGB1) and soluble triggering receptor-1 (sTREM-1) levels in the prognostic assessment of trauma

**DOI:** 10.5937/jomb0-59276

**Published:** 2026-01-28

**Authors:** Lulu Tang, Dan Shan, Heng Zhang, Shuli Lin, Xubiao Ji

**Affiliations:** 1 Aoyang Hospital Affiliated to Jiangsu University, Department of Orthopaedics, China; 2 Huai'an Clinical Medical College of Jiangsu University (Huai'an Hospital of Huai'an City), Department of Operating Room, Huai'an District, China; 3 Qilu Hospital of Shandong University, Department of Trauma Orthopaedics, China; 4 Changzhou Wujin People's Hospital (Wujin Hospital Affiliated with Jiangsu University), Department of Spinal Surgery, China

**Keywords:** multiple injuries, high mobility group protein B1, soluble triggering receptor-1 of myeloid cells, prognosis assessment, visestruke povrede, protein visoke pokretljivosti grupe B1, rastvorljivi receptor za aktivaciju mijeloidnih celija 1, procena prognoze

## Abstract

**Background:**

This study analysed the clinical value of serum high-mobility group protein B1 (HMGB1) and soluble triggering receptor expressed on myeloid cells 1 (sTREM-1) in the prognostic assessment of trauma patients.

**Methods:**

This prospective cohort study included 92 patients with multiple injuries admitted to our hospital between December 2022 and December 2024. The patients at admission were divided into three groups according to their Injury Severity Score: the minor injury group (n=24), the moderate injury group (n = 58), and the severe injury group (n = 10). The patients were divided into the MODS group (n=20) and the non-MODS group (n=72) on the basis of whether they had multiple organ dysfunction syndrome (MODS) after admission. The patients were divided into a death group (n = 13) and a survival group (n=79) on the basis of their outcomes within 28 days after the occurrence of trauma. Venous blood was collected from an empty stomach at 24 hours, 72 hours and 7 days after injury. The levels of serum HMGB1 and sTREM-1 were determined using an enzyme-linked immunosorbent assay (ELISA). Moreover, the injury severity score (ISS), Acute Physiology and Chronic Health Evaluation (APACHE II), complications during hospitalisation (infection, MODS, etc.) and 28-day survival of the patients were recorded.

**Results:**

The concentrations of serum HMGB1 and sTREM-1 in the trauma group were significantly greater than those in the control group (P&lt; 0.01) and increased with increasing ISS. The peak levels of HMGB1 and sTREM-1 in the poor-prognosis group (death/complications) were significantly higher than those in the good-prognosis group (P&lt; 0.001). The predictive efficacy (AU C= 0.891) of the combined detection of dual indicators for post-traumatic complications was greater than that of the single indicators (AU C= 0.812 for HMGB1 and A U C= 0.784 for sTREM-1), and the area under the ROC curve for the 28-day risk of death reached 0.927. Multivariate logistic regression analysis confirmed that both factors were independent risk factors for trauma prognosis (O R= 3.42 and O R= 2.98, respectively).

**Conclusions:**

HMGB1 and sTREM-1 significantly increase in the early stage of trauma and are closely related to the severity of injury and poor prognosis. Combined dynamic monitoring can effectively predict complications and the risk of mortality, providing a crucial biomarker basis for early clinical intervention.

## Abstract (sr)

**Uvod:**

Ova studija je analizirala klinički značaj serumske koncentracije proteina visoke pokretljivosti grupe B1 (HMGB1) i rastvorljivog receptora za aktivaciju na mijeloidnim celijama 1 (sTREM-1) u proceni prognoze kod pacijenata sa traumom.

**Metode:**

Ova prospektivna kohortna studija je obuhvatila 92 pacijenta sa visestrukim povredama, primljenih u nasu bolnicu u periodu od decembra 2022. do decembra 2024. godine. Na osnovu Skopa težine povreda (Injury Severity Score - ISS), pacijenti su po prijemu podeljeni u tri grupe: grupa sa laksim povredama (n= 24), grupa sa umerenim povredama (n = 58) i grupa sa teskim povredama (n = 10). Prema prisustvu sindroma disfunkcije vise organa (MODS) nakon prijema, pacijenti su podeljeni u MODS grupu (n=20) i grupu bez MODS-a (n=72). Na osnovu ishoda u roku od 28 dana nakon traume, pacijenti su svrstani u grupu preminulih (n = 13) i grupu preživelih (n=79). Venska krv je uzeta nataste 24 sata, 72 sata i 7 dana nakon povrede. Nivoi HMGB1 i sTREM-1 u serumu određeni su pomoću testa enzimski vezanog imunosorbensa (ELISA).

**Rezultati:**

Koncentracije HMGB1 i sTREM-1 u serumu kod pacijenata sa traumom bile su značajno više nego u kontrolnoj grupi (P&lt; 0,01) i povećavale su se sa porastom ISS-a. Maksimalni nivoi HMGB1 i sTREM-1 u grupi sa lošom prognozom (smrt/komplikacije) bili su značajno viši nego u grupi sa dobrom prognozom (P &lt; 0 ,0 01). Prediktivna efikasnost kombinovanog određivanja oba biomarkera za posttraumatske komplikacije bila je veća (AU C= 0,891) nego kod pojedinačnih markera (AU C= 0,812 za HMGB1 i A U C = 0,784 za sTREM-1), dok je površina ispod ROC krive za procenu rizika smrtnosti u roku od 28 dana dostigla 0 ,927 . Multivarijantna logistička regresiona analiza potvrdila je da su oba markera nezavisni faktori rizika za prognozu kod traume (O R= 3,42 i O R= 2,98).

**Zaključak:**

Nivoi HMGB1 i sTREM-1 se značajno povećavaju u ranom stadijumu traume i usko su povezani sa težinom povreda i lošom prognozom. Kombinovano dinamičko praćenje može efikasno predvideti komplikacije i rizik od smrtnog ishoda, predstavljajući važnu osnovu za ranu kliničku intervenciju.

## Introduction

Multiple trauma refers to trauma to two or more tissues or organs caused by one pathogenic factor. It is characterised by severe trauma, rapid changes in condition and easy progression, and the risk of death for patients is relatively high [1] [2]. Accurately assessing the severity and prognosis of the disease, and making informed clinical decisions, are crucial to reducing complications and mortality rates, as well as improving patient outcomes. Studies [3] [4] have confirmed that patients with multiple types of trauma have elevated systemic inflammatory levels, which are related to the progression of multiple types of trauma. High mobility group box 1 (HMGB1) is a protein produced after macrophages are stimulated by the lipopolysaccharide, interleukin and tumour necrosis factor α (TNF-α) of gram-negative bacteria. It is an essential late inflammatory mediator [5]. Soluble triggering receptor expressed on myeloid cells-1 (sTREM-1) is an inflammatory response-triggering receptor that has been discovered in recent years and has also been confirmed to play a crucial role in the process of inflammatory triggering and amplification [6]. At present, there is evidence [7] that HMGB1 is correlated with sTREM-1. Moreover, other studies [8] [9] have shown that the levels of HMGB1 and sTREM-1 in patients with multiple injuries are significantly increased and that patients with high levels of HMGB1 and sTREM-1 have a poor prognosis. However, studies on the changes in HMGB1 and sTREM-1 levels at different time points after trauma in patients with multiple injuries and their correlations with the severity of the disease, complications and death are rare, and the relevant conclusions are not clear enough.

Therefore, in this study, by detecting the levels of serum HMGB1 and TREM-1 in patients with multiple injuries at 24 h, 72 h and 7 d after trauma occurrence, the correlations between the changes in HMGB1 and sTREM-1 in patients and the severity of the disease, complications and death were analysed, with the aim of providing a basis for the judgment of patients' conditions, prognosis assessment and research on intervention measures. These findings can be used to inform reasonable clinical decisionmaking and improve patient prognosis.

## Materials and methods

### Research subjects

A total of 92 patients with multiple injuries who were admitted to the emergency department of our hospital between December 2022 and December 2024 were selected as research subjects.

The inclusion criteria for patients were as follows: ① aged 18-65 years; ② met the diagnostic criteria for multiple injuries in »History and Diagnosis of Multiple Injuries: Expert Consensus Opinion (2023 Edition)« [10]; ③ the time from the occurrence of trauma to admission did not exceed 6 hours; ④ the survival time after admission was >7 days, and there was a maximum follow-up record of 28 days; and ⑤ patients or their families provided informed consent for this study. The exclusion criteria for patients were as follows: ① had concurrent infectious diseases, immune diseases, etc.; ② had concurrent malignant tumours; ③ were pregnant or lactating; and ④ were transferred to another hospital halfway through or stopped treatment. The patients were divided into three groups according to their ISS at admission [11]: the minor injury group (ISS<16, n = 24), the severe injury group (16 ISS 25, n = 58), and the severe injury group (ISS>25, n = 10). The patients were divided into the MODS group (n = 20) and the non-MODS group (n = 72) according to whether multiple organ dysfunction syndrome (MODS) was present after admission [12]. The patients were divided into a death group (n = 13) and a survival group (n = 79) on the basis of their outcomes within 28 days after the occurrence of trauma.

### Data collection

The general and clinical data of the patients, including age, sex, presence or absence of underlying diseases, time of admission, injury factors, main trauma sites and quantities, whether emergency surgery was performed, and whether large-scale blood transfusion was carried out, were collected. The occurrence of MODS in patients during hospitalisation and survival within 28 days after trauma were statistically analysed.

### ISS and Glasgow Coma Scale

Immediately after admission, the ISS and Glasgow Coma Scale (GCS) score were evaluated. ISS score: The human body is divided into six parts: head and neck, face, chest, abdomen, limbs/pelvis, and body surface. The score of each part is calculated according to the abbreviated injury scale (AIS) (1 to 5 points). The sum of the squares of the three AIS values with the highest scores is the ISS score, with a maximum of 75 points. The GCS score is scored and graded based on different aspects of eye-opening response, language response, and motor response, with a maximum of 15 points.

### Index detection

Venous blood was collected within 2 hours after patient admission. The levels of activated partial thromboplastin time (APTT) and D-dimer (DD) in the patients were detected via an automatic blood coagulation analyser. The level of hypersensitive C-reactive protein (hs-CRP) was measured using a fully automated biochemical analyser. The level of procalcitonin (PCT) was detected via the double-antibody sandwich immunochemiluminescence method. Venous blood was drawn from the patients at 24 h, 72 h and 7 d after the trauma occurred. The serum was collected by centrifugation and stored at -80°C. The levels of serum HMGB1, sTREM-1 and interleukin-6 (IL-6) were detected via an enzyme-linked immunosorbent assay (ELISA) kit (Beijing Solebao Technology Co., Ltd.).

### Statistical analysis

Statistical processing was performed via SPSS 19.0 software. Quantitative data following a normal distribution are expressed as x̄±s. Independent sample t-tests were used for comparisons between two groups, one-way analysis of variance was used for comparisons among multiple groups, and the least significant difference (LSD) t-test was used for pairwise comparisons between groups. Qualitative data are expressed as frequencies (percentages), and comparisons between groups were performed via Fisher's exact probability method. Repeated-measure analysis was employed for the comparison of the same indicator at different time points. Correlation analysis was conducted via Pearson correlation. Multivariate logistic regression analysis was used to analyse the factors influencing adverse outcomes. The predictive value of HMGB1 and sTREM-1 for adverse outcomes was evaluated via receiver operating characteristic (ROC) curves. P<0.05 indicated a statistically significant difference.

## Results

### Changes in HMGB1 and sTREM-1 levels in patients with multiple injuries of different severities

The levels of HMGB1 and sTREM-1 in patients with multiple injuries of varying severities changed over time (all P<0.05). The level of HMGB1 increased over time, and the level of sTREM-1 initially increased but then decreased. The levels of HMGB1 and sTREM-1 in the severe injury and severe injury groups at 24 h, 72 h and 7 d after trauma were significantly greater than those in the minor injury group, and the differences were statistically significant (all P<0.05). The levels of HMGB1 at 72 hours and 7 days after trauma and the levels of sTREM-1 at 24 hours and 72 hours in the severe injury group were significantly greater than those in the severe injury group, and the differences were statistically significant (all P<0.05). The results are shown in Table 1.

**Table 1:** Comparison of HMGB1 and sTREM-1 levels in patients with multiple injuries of different severity.

Variable	Light injury group<br>(n=24)	Grave injury group<br>(n = 58)	Severe injury group<br>(n = 10)	P value	P value	P value
HMGB1/(ng·mL^-1^)						
24 h	2.47±0.61	2.86±0.65	2.99±0.52	0.013	0.03	0.531
72 h	3.12±0.73	3.57±0.66	4.22±0.31	0.006	0	0.005
7 d	3.63±0.83	4.19±0.77	4.96±0.35	0.003	0	0.004
sTREM-1/(ng·mL^-1^)						
24 h	11.71±3.00	13.43±2.65	15.38±1.95	0.01	0	0.037
72 h	12.77±3.47	14.46±2.74	16.51±1.99	0.017	0.001	0.041
7 d	5.52±1.24	6.47±1.25	6.72±1.20	0.006	0.015	0.463

### Correlation analysis of HMGB1 and sTREM-1 levels

**Figure 1:**
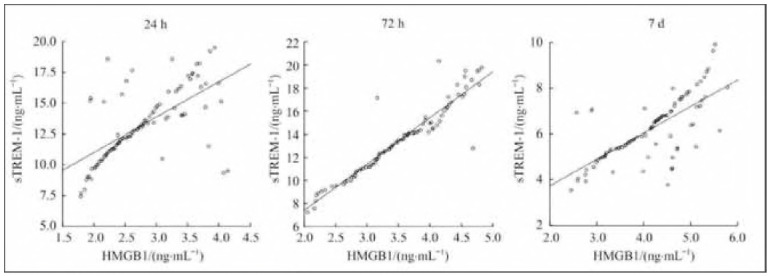
Correlation analysis of the levels of HMGB1 and sTREM-1

The results of the correlation analysis revealed that the levels of HMGB1 and sTREM-1 at each time point were significantly positively correlated (r= 0.645, r=0.942, r=0.722; P=0.000). The results are shown in Figure 1.

### Changes in HMGB1 and sTREM-1 levels in patients with multiple injuries in the MODS group and the non-MODS group

The levels of HMGB1 and sTREM-1 in both the MODS group and the non-MODS group changed over time (P<0.05). The level of HMGB1 increased over time, and the level of sTREM-1 initially increased but then decreased. The levels of HMGB1 at 72 h and 7 d and the levels of sTREM-1 at 24 h and 72 h after trauma in the MODS group were significantly greater than those in the non-MODS group (P=0.011, P=0.008, P=0.020, P=0.014). The results are shown in Table 2.

**Table 2:** Comparison of HMGB1 and sTREM-1 levels between the MODS group and the non-MODS group.

Variable	Non-MODS group (n=72)	MODS group (n=20)	P value
HMGB1/(ng·mL<^-1^)			
24 h	2.71±0.65	2.99±0.60	0.086
72 h	3.42±0.70	3.88±0.66	0.011
7 d	4.01±0.82	4.57±0.76	0.008
sTREM-1/(ng·mL^-1^)			
24 h	12.83±2.77	14.50±2.87	0.02
72 h	13.84±2.96	15.71±2.97	0.014
7 d	6.07±1.20	6.69±1.71	0.069

### Relationships between the levels of HMGB1 and sTREM-1 and the prognosis of patients with multiple injuries

### Comparison of basic data between the survival group and the nonsurviving group

There were statistically significant differences in the comparison of admission time, distribution of major trauma sites, disease severity, MODS incidence, and levels of hs-CRF, IL-6, and PCT between the death group and the survival group (all P < 0.05). In contrast, there were no statistically significant differences in other data between the two groups (P>0.05). The results are shown in Table 3.

**Table 3:** Comparison of basic data between the survival group and the death group.

Variable	Survival group (n=79)	Death group (n = 13)	t/x^2 ^value	P value
Age/year	42.53±11.64	47.31±11.71	1.369	0.174
Gender/n (%)			0.837	0.546
Male	44 (55.70)	9 (69.23)		
Female	35 (40.30)	4 (30.77)		
Background disease/n (%)			0.387	0.747
Yes	25 (31.65)	3 (23.08)		
No	54 (68.35)	10 (76.92)		
Admission time/h	4.43±0.92	5.00±0.51	3.259	0.003
Injury factor/n (%)			1.224	0.57
Transportation	54 (68.35)	8 (61.54)		
Fall accident from a high place	19 (24.05)	3 (23.08)		
Other	6 (7.59)	2 (15.38)		
Number of wound sites/n	2.66±0.64	2.92±0.49	1.425	0.158
Primary trauma site/n (%)			7.4	0.042
Head	32 (40.51)	11 (84.62)		
Chest	27 (34.18)	1 (7.69)		
Abdomen	14 (17.72)	1 (7.69)		
Limbs and pelvis	6 (7.59)	0 (0)		
Emergency operation/n (%)			2.391	0.181
Yes	20 (25.32)	6 (46.15)		
No	59 (74.68)	7 (53.85)		
Massive transfusion/n (%)			4.778	0.055
Yes	24 (34.18)	8 (61.54)		
No	55 (65.82)	5 (38.46)		
Severity/n (%)			19.475	0
Lightly injury	22 (27.85)	2 (15.38)		
Gravely injury	54 (68.35)	4 (30.77)		
Severe injury	3 (3.80)	7 (53.85)		
GCS score/score	10.31±2.04	9.51±2.27	1.286	0.202
APTT/s	30.31±5.02	32.97±5.91	1.727	0.088
DD/(mg-L^-1^)	0.81±0.37	0.89±0.29	0.77	0.443
MODS/n (%)			5.304	0.032
Yes	14 (17.72)	6 (46.15)		
No	65 (82.28)	7 (53.85)		
hs-CRP/(mg·L^-1^)	18.74±3.98	21.79±3.28	2.612	0.011
IL-6/(pg·mL^-1^)	65.16±14.11	78.33±10.46	3.215	0.002
PCT/(ng·mL^-1^)	4.06±0.90	5.02±0.91	3.56	0.001

### Changes in HMGB1 and sTREM-1 levels in the survival group and the death group

The levels of HMGB1 and sTREM-1 in both patient groups changed over time (P<0.05). The level of HMGB1 increased over time, and the level of sTREM-1 initially increased but then decreased. The levels of HMGB1 at 72 h and 7 d and sTREM-1 at 24 h and 72 h after trauma in the death group were significantly greater than those in the survival group, and the differences were statistically significant (all P=0.000). The results are shown in Table 4.

**Table 4:** Comparison of HMGB1 and s TREM-1 levels between the survival group and the death group.

Variable	Survival group (n=79)	Death group (n=13)	P value
HMGB1/(ng·mL^-1^)			
24 h	2.73±0.65	3.05±0.57	0.096
72 h	3.40±0.69	4.23±0.43	0
7 d	3.97±0.79	5.07±0.44	0
sTREM-1/(ng·mL^-1^)			
24 h	12.78±2.75	15.70±2.21	0
72 h	13.68±2.79	17.66±2.23	0
7 d	6.16±1.30	6.92±1.20	0.106

### Multivariate logistic regression analysis of survival outcomes in patients with multiple injuries

The survival of patients within 28 days after trauma was used as the dependent variable, and the admission time, leading trauma site, severity of the disease, levels of hs-CRP IL-6, and PCT, and presence of MODS, as well as the levels of HMGB1 at 72 hours and 7 days after trauma and sTREM-1 at 24 hours and 72 hours, were used as independent variables. Multivariate logistic regression analysis was conducted via the »conditional forward« method. The results revealed that the level of HMGB1 7 days after trauma (OR=35.600, P=0.011), the time of admission (OR=3.743, P=0.042), and the level of hs-CRP (OR=1.516, P=0.004) were independent risk factors for death 28 days after trauma in patients with multiple injuries. The results are shown in Table 5.

**Table 5:** Multivariate Logistic regression analysis of survival outcomes in patients with multiple injuries.

Variable	B	Wals	Sig	OR	95% CI
Admission time	1.32	4.136	0.042	3.743	1.049-13.358
hs-CRP	0.416	8.247	0.004	1.516	1.141-2.014
HMGB1^7 d^	3.572	12.131	0.011	35.6	4.769-265.752

### ROC curve analysis

**Figure 2:**
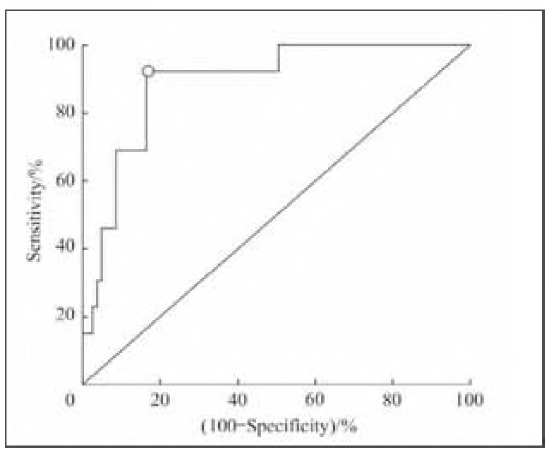
Serum HMGB1 level ROC curve analysis for the prediction of 28-day mortality in patients with multiple injuries.

The results of multivariate logistic regression analysis revealed that the level of HMGB1 7 days after trauma was an independent risk factor for death 28 days after trauma in patients with multiple injuries. Therefore, the ROC curve was used to further analyse the predictive value of this index for death 28 days after trauma. The area under the curve (AUC) of the HMGB1 level 7 days after trauma for predicting 28-day death was 0.890 (95% CI, 0.808-0.946). The cut-off value was 4.66 ng/mL, with a sensitivity of 83.5% and a specificity of 92.3%. The results are shown in Figure 2.

## Discussion

Multiple injuries are characterised by severe injury severity, rapid changes in injury conditions, high infection and mortality rates, and complex causes of death. They have gradually become one of the main causes of clinical death [13]. Therefore, early detection, identification, and provision of active and effective treatment, as well as prevention of related complications, are key to reducing the mortality rate. Although many biochemical indicators for evaluating the severity and prognosis of multiple injuries have been identified in clinical practice, their early diagnostic sensitivity is low, and their specificity is not high. Therefore, new indicators are needed that can more accurately determine the severity of injury and prognosis for patients with multiple injuries in the early stages of the disease.

HMGB-1 is an important chromatin-binding protein involved mainly in DNA replication, gene transcription regulation, stabilising chromatin structure, and cell differentiation [14] [15]. Relevant studies [16] [17] have confirmed that HMGB-1 plays a crucial role in the inflammatory response process of systemic inflammatory diseases, autoimmune diseases, and inflammatory diseases affecting tissues and organs, and is an active mediator of late inflammatory responses. Current studies [18] [19] have shown that the plasma HMGB1 concentration in patients with traumatic injury is significantly increased, which is helpful in determining the prognosis of patients. Studies [20] have shown that the level of HMGB-1 in trauma patients is significantly greater than that in healthy individuals and is associated with the risk of in-hospital mortality. Another study [21] reported that the serum HMGB-1 levels of MODS patients with acute multiple abdominal injuries within one day of admission were significantly higher than those of non-MODS patients. The results of this study revealed that the HMGB-1 level in patients with MODS at different time points after trauma was greater than that in non-MODS patients. Moreover, the HMGB1 levels at 72 h and 7 d and the sTREM-1 levels at 24 h and 72 h after trauma in the death group were greater than those in the survival group, suggesting that the HMGB-1 level may be related to complications and prognosis. This finding is consistent with the results of previous studies. The results of multivariate logistic regression analysis revealed that the HMGB1 level 7 days after trauma was an independent influencing factor for death 28 days after trauma in patients with multiple injuries, indicating that it was closely related to the adverse outcomes of patients with multiple injuries.

Further ROC curve analysis revealed that the AUC of the HMGB-1 level 7 days after trauma for the prediction of death within 28 days was 0.890, with a sensitivity of 83.5% and a specificity of 92.3%, which was greater than the value of the IL-6 level for the prediction of survival in patients with multiple abdominal injuries (AUC=0.780, sensitivity of 79.2%, specificity of 79.6%) [21]. These findings suggest that the HMGB-1 level 7 days after trauma has high predictive value for the adverse outcomes of patients with multiple injuries. This may be because HMGB1 is a potential late-phase inflammatory mediator. Compared to early inflammatory mediators (such as CRP IL-1, and IL-6), the level of HMGB1 changes later and persists longer. Therefore, from the perspective of the detection time window, it may be more suitable for assessing the condition of trauma patients [22]. Previous studies [21] [23] have demonstrated that serum HMGB-1 levels possess predictive value for the occurrence of MODS and patient survival outcomes. These results indicate that the HMGB-1 level 7 days after trauma can be used as a biomarker for prognosis assessment in patients with multiple injuries.

sTREM-1 is a newly discovered inflammatory response-triggering receptor. It is a secretory subtype lacking transmembrane domains and plays a crucial role in the diagnosis of infectious diseases, such as sepsis, ventilator-associated pneumonia, and bacterial meningitis [24] [25]. Current studies [26] [27] have shown that sTREM-1 is closely related to traumatic injury, the severity of systemic inflammatory response syndrome, and the occurrence and development of sepsis. Another study [28] reported that the serum sTREM-1 level within one week after injury in patients with severe multiple injuries combined with MODS was significantly higher than that in patients without MODS, and the sTREM-1 level in deceased patients was higher than that in survivors. The results of this study show that the level of sTREM-1 in patients with multiple injuries increases 24 hours after admission, peaks 72 hours after the trauma occurs, and then tends to decrease. During this process, the level of sTREM-1 in patients with MODS was greater than that in non-MODS patients at different time points after trauma. The sTREM-1 levels in the nonsurviving group were greater than those in the surviving group. The above results suggest that the elevated level of sTREM-1 may be related to complications and prognosis [29]. However, the results of multivariate logistic regression analysis revealed that sTREM-1 levels at different time points were not independent influencing factors for 28-day death after trauma in patients with multiple injuries.

## Conclusion

The levels of serum HMGB1 and sTREM-1 at different time points after trauma in patients with multiple injuries are correlated with MODS and survival outcomes. The level of HMGB-1 at 7 days after trauma can be used as a biomarker for prognosis evaluation in patients with multiple injuries. However, the sample size of this study was small, and it was conducted at a single centre. Therefore, a study with a larger sample size is needed to confirm the correlation between the levels of serum HMGB1 and sTREM-1 in patients with multiple types of trauma and MODS, as well as to investigate whether the two have combined predictive value for survival outcomes. Moreover, further basic research is needed to clarify the mechanism by which HMGB1 and sTREM-1 are involved in the progression of multiple injuries.

## Dodatak

### Authors' contributions

Lulu Tang is the first author of the study, with Dan Shan as co-first author, who contributed equally to the research.

### Conflict of interest statement

All the authors declare that they have no conflict of interest in this work.
